# Crystal structure of 3-benzamido-1-(4-nitro­benz­yl)quinolinium tri­fluoro­methane­sulfonate

**DOI:** 10.1107/S2056989016006423

**Published:** 2016-04-29

**Authors:** Mariana Nicolas-Gomez, Iván J. Bazany-Rodríguez, Eduardo Plata-Vargas, Simón Hernández-Ortega, Alejandro Dorazco-González

**Affiliations:** a Centro Conjunto de Investigacion en Quimica Sustentable UAEM–UNAM, Instituto de Quimica, Universidad Nacional Autonoma de Mexico, Carretera Toluca-Atlacomulco, Km 14.5 CP 50200 Toluca, Estado de Mexico, Mexico; b Laboratorio de Rayos-X, Instituto de Quimica, UNAM, Circuito Exterior, Ciudad Universitaria Deleg. Coyoacán México 04510, México DF, México, Mexico

**Keywords:** crystal structure, benzamide, tri­fluoro­methane­sulfonate salt, *p*-nitro­benzyl­quinolinium

## Abstract

In the title compound, C_23_H_18_N_3_O_3_^+^·CF_3_SO_3_^−^, the asymmetric unit contains two crystallographically independent organic cations with similar conformations. Each cation shows a moderate distortion between the planes of the amide groups and the quinolinium rings with dihedral angles of 14.90 (2) and 31.66 (2)°. The quinolinium and phenyl rings are slightly twisted with respect to each other at dihedral angles of 6.99 (4) and 8.54 (4)°. The tri­fluoro­methane­sulfonate anions are linked to the organic cations *via* N—H⋯O hydrogen-bonding inter­actions involving the NH amide groups. In the crystal, the organic cations are linked by weak C—H⋯O(nitro group) inter­actions into supramol­ecular chains propagating along the *b*-axis direction.

## Abstract 2

In the title salt, each cation shows a moderate distortion between the planes of the amide groups and the quinolinium rings. The tri­fluoro­methane­sulfonate anions are linked to organic cations *via* N—H⋯O hydrogen-bonding inter­actions involving the NH amide groups. In the crystal, weak C—H⋯O hydrogen bonds and π-stacking inter­actions between the quinolinium and phenyl rings link the organic cations into chains.

## Chemical context   

Quinoline-based quaternary salts have attracted the attention of researchers in different areas of organic chemistry for their relevant applications such as DNA-inter­calators (Mazzoli *et al.*, 2011 ▸), fluorescent pH-sensors (Badugu *et al.*, 2005*a* ▸), fluorescent labels for anti­biotics (Zeng *et al.*, 2010 ▸), proteins (Hong *et al.*, 2004 ▸), heparin (Sauceda *et al.*, 2007 ▸), sacharides (Badugu *et al.*, 2005*b* ▸), fluorescent probes for fluoride and cyanide ions (Badugu *et al.*, 2004 ▸) and nucleotides (Dorazco-González *et al.*, 2014 ▸). These cationic organic compounds are probably the most used fluorescent sensors for chloride ions in aqueous media (Bazany-Rodríguez *et al.*, 2015 ▸) and intra­cell samples (Baù *et al.*, 2012 ▸). On the other hand, benzamide compounds are used as inter­mediaries for the synthesis of species with biological activity such as 1,4-benzodiazepinones, thia­zoles and oxazoles (Majumdar & Ganai, 2011 ▸; Majumdar & Ghosh, 2013 ▸; Majumdar *et al.*, 2012 ▸) and bicyclic *N*-hetero­cycles and nitro­gen-rich medium-size heterocycles (Mondal *et al.*, 2012 ▸; Zeni & Larock, 2006 ▸; Ohta *et al.*, 2008 ▸; Majumdar *et al.*, 2008 ▸; Raju *et al.*, 2009 ▸; Evdokimov *et al.*, 2011 ▸).

## Structural commentary   

**Figure 1:**
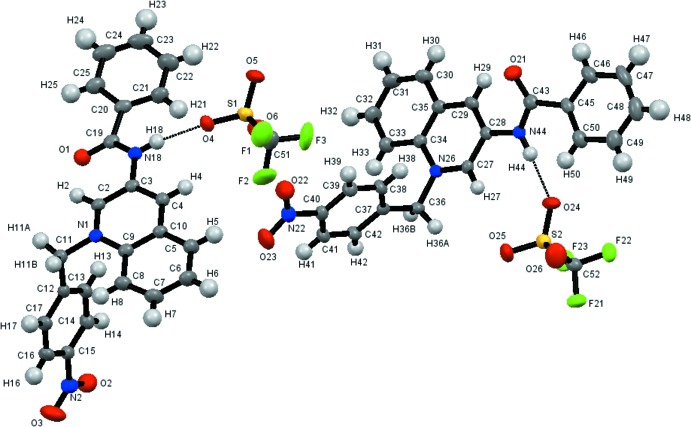
The asymmetric unit of the title compound, showing the atom labelling. Displacement ellipsoids are drawn at the 30% probability level. Hydrogen bonds are shown as dashed lines. [Symmetry codes: (A) *x*, −*y* + 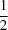, *z* + 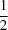; (B) *x* − 1, *y*, *z* + 1.]

The asymmetric unit of the title compound comprises two independent organic [*N*-[3-*N′*-(*p*-nitro­benz­yl)quinolinium]benzamide] cations, each of which is linked to one triflate anion through hydrogen-bonding inter­actions (N—H⋯O) between the amide groups and anions (Figs. 1 ▸ and 2 ▸; Table 1 ▸). Each cation shows a distortion between the mean planes of the amide groups and the quinolinium rings, with dihedral angles of 14.90 (2) and 31.66 (2)°. The phenyl and quinolinium rings are practically coplanar with dihedral angles of 6.99 (4) and 8.54 (4)°.
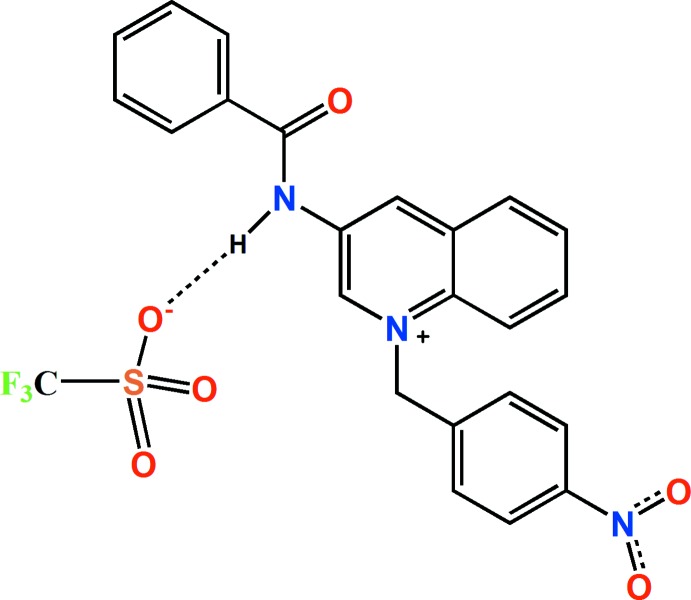


## Supra­molecular features   

**Figure 3:**
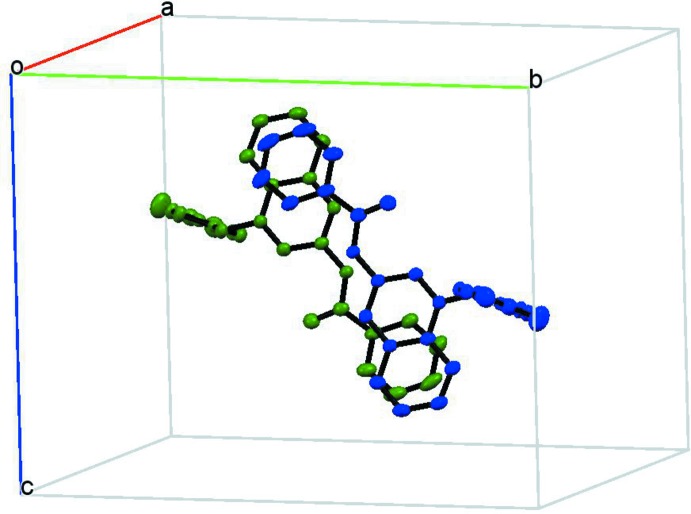
A view approximately along the *a* axis, showing the offset face-to-face π-inter­actions between the benzamide and the quinolinium group. H atoms and tri­fluoro­methane­sulfonate anions have been omitted for clarity.

The supra­molecular structure involves triflate ion pairing with the bulky cation *via* N—H⋯O hydrogen bonds (Table 1 ▸) between amide groups and anions. The crystal structure also features face-to-face π-stacking inter­actions between benzamide and quinolinium rings [inter-centroid distance, 3.71 (3) Å] forming chains along the *b*-axis direction, as shown in Figs. 3 ▸ and 4 ▸. The triflate anions are located on the periphery of the quinolinium groups, establishing C—H⋯O interactions (Table 1 ▸).

## Database survey   

A search of the Cambridge Structural Database (CSD, Version 35.6, last update 2015; Groom *et al.*, 2016 ▸) using *N*-(naphthalen-3-yl)benzamide as the main structure, reveals 26 hits; however using a closer structure, *N*-(quinolin-3-yl)benz­amide, shows only one hit, which corresponds to the triflate salt of *N*-(3-*N′*-methyl­quinolinium)benzamide (RISQEP) (Dorazco-González *et al.*, 2014 ▸). Additionally, *N*-methyl­ated and benzyl­ated isomers were found; *N*-(5-*N′*-methyl­quino­linium)benzamide triflate and *N*-(6-*N′*-methyl­quinolinium)benzamide triflate (RISQOB and RISQIV, respectively; Dorazco-González *et al.*, 2014 ▸) and *N*-(6-*N′*-benzyl­quino­linium)benzamide bromide (AJEREO;Bazany-Rodríguez *et al.*, 2015 ▸). On the other hand, the related (1,10-phenanthrolin-5-yl)benzamide Ir^III^ complex (FAPLEP; Castillo *et al.*, 2012 ▸) and Ru^II^ and Re^I^ complexes containing the chemical fragment *N*-(quinolin-3-yl)benzamide (NILFAQ and NILFEU; Batey *et al.*, 2007 ▸) have been reported previously as luminescent chemosensors. The structure of *N*-(1,10-phenanthrolin-5-yl)-4-(2-pyrid­yl(benzamide) monohydrate (ROFTOW; Kobayashi *et al.*, 2008 ▸) has also been reported.

## Synthesis and crystallization   

A mixture of 6-amino­quinoline (1.0 g, 6.9 mmol) and benzoyl chloride (0.49 g, 3.45 mmol) in 30 mL of dry toluene-acetone (1:1 *v/*v) was stirred under reflux for 2.5 h. The white precipitate was collected by filtration and washed with acetone and 5% NaHCO_3_ to give *N*-(3-quinolin­yl)benzamide in 90% yield, which was reacted with 1.5 equiv. of *p*-nitro benzyl chloride in 30 mL of dry DMF for 5 h. The resulting yellow powder was filtered and washed with cold MeOH to give the chloride salt in 85% yield. The chloride salt was dissolved in 100 mL of hot H_2_O-MeOH (1:1 *v*/*v*) then one equiv. of silver triflate was added, the mixture was stirred at room temperature for 4 h. The precipitate of silver chloride was filtered off and yellow crystals were obtained by evaporation of the solvent at room temperature.

^1^H NMR (300MHz, DMSO-*d_6_*) δ 11.42 (*s*, 1H), 10.08 (*s*, 1H), 9.51 (*s*, 1H), 8.55 (*d*, 1H), 8.42 (*d*, 1H), 8.29 (*d*, 2H), 8.10 (*d*, 2H), 7.97 (*t*, 1H), 7.73 (*m*, 6H), 6.63 (*s*, 2H). IR (ATR) cm^−1^ 3271.41 (*d*), 3073.52 (*d*), 2993.15 (*d*), 1685.80 (*d*), 1603.94 (*d*), 1551.56 (*m*), 1518.86 (*f*), 1490.75 (*d*), 1372.93 (*m*), 1344.45 (*f*), 1272.46 (*f*), 1251.94 (*f*), 1163.90 (*f*), 1107.99 (*d*), 1028.82 (*f*), 900.95 (*d*), 847.43 (*d*), 800.46 (*d*), 760.49 (*d*), 741.83 (*m*), 710.10 (*m*), 693.06 (*m*), 665.58 (*d*), 633.92 (*f*), 573.28 (*d*), 515.54 (*m*), 434.41 (*m*).

## Refinement   

Crystal data, data collection and structure refinement details are summarized in Table 2 ▸. All non-hydrogen atoms were refined anisotropically. H atoms attached to C atoms were placed in geometrically idealized positions and constrained to ride on their parent atoms, with C—H = 0.93–0.98 Å and *U*_iso_(H) = 1.2*U*_eq_(C) for aromatic groups and *U*_iso_(H) = 1.5 *U*_eq_(C) for aliphatic groups (Sheldrick, 2008 ▸). N—H hydrogen atoms were localized in difference Fourier maps and refined with the bond lengths fixed at 0.90 A and the isotropic temperature factors fixed at 1.2 times those of the corres­ponding nitro­gen atom.

## Supplementary Material

Crystal structure: contains datablock(s) I. DOI: 10.1107/S2056989016006423/hg5469sup1.cif

Structure factors: contains datablock(s) I. DOI: 10.1107/S2056989016006423/hg5469Isup2.hkl

Supporting information file. DOI: 10.1107/S2056989016006423/hg5469Isup3.cml

CCDC reference: 1474439

Additional supporting information:  crystallographic information; 3D view; checkCIF report

## Figures and Tables

**Figure 2:**
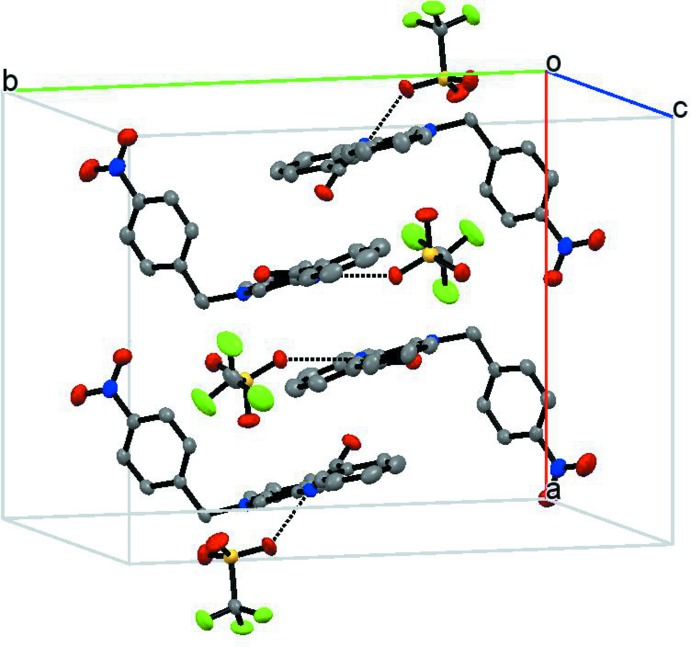
Perspective view of a fragment of the crystal structure of the title compound with hydrogen bonds N—H⋯O shown as dashed lines. Displacement ellipsoids are drawn at the 30% probability level. H atoms have been omitted for clarity.

**Figure 4:**
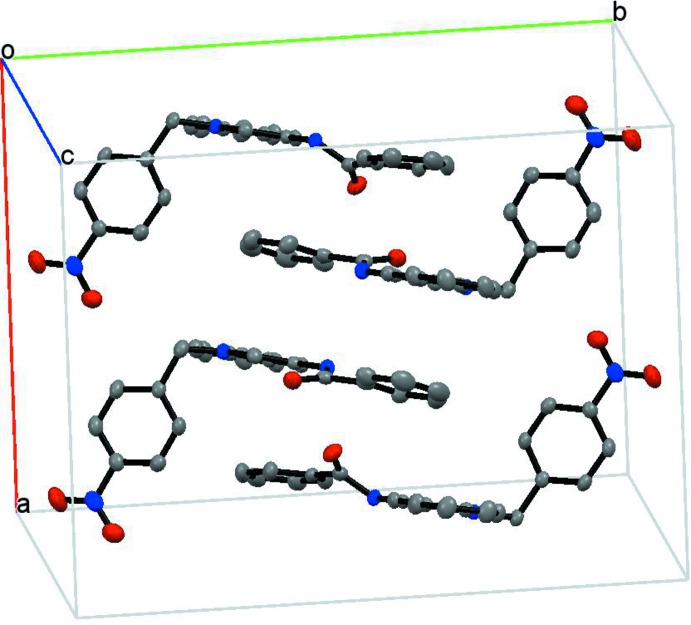
View of the π-aggregated structure. Hydrogen atoms and tri­fluoro­methane­sulfonate anions have been omitted for clarity.

**Table 1:** Hydrogen-bond geometry (Å, °)

*D*—H⋯*A*	*D*—H	H⋯*A*	*D*⋯*A*	*D*—H⋯*A*
N18—H18⋯O4^i^	0.88 (3)	2.15 (3)	2.982 (3)	156 (3)
N44—H44⋯O24^ii^	0.87 (2)	1.97 (3)	2.811 (3)	164 (3)

Symmetry codes: (i) 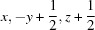; (ii) 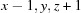.

**Table 2:** Experimental details

Crystal data	
Chemical formula	C_23_H_18_N_3_O_3_^+^·CF_3_O_3_S^−^
*M*_r_	533.47
Crystal system, space group	Monoclinic, *P*2_1_/*c*
Temperature (K)	298
*a*, *b*, *c* (Å)	15.2183 (6), 20.0810 (8), 15.3652 (6)
β (°)	90.544 (1)
*V* (Å^3^)	4695.4 (3)
*Z*	8
Radiation type	Mo *K*α
μ (mm^−1^)	0.21
Crystal size (mm)	0.30 × 0.17 × 0.14

Data collection	
Diffractometer	Bruker APEXII CCD area-detector
No. of measured, independent and observed [*I* > 2σ(*I*)] reflections	38624, 8609, 3809
*R*_int_	0.095
(sin θ/λ)_max_ (Å^−1^)	0.603

Refinement	
*R*[*F* ^2^ > 2σ(*F* ^2^)], *wR*(*F* ^2^), *S*	0.046, 0.110, 0.85
No. of reflections	8609
No. of parameters	729
No. of restraints	180
H-atom treatment	H atoms treated by a mixture of independent and constrained refinement
Δρ_max_, Δρ_min_ (e Å^−3^)	0.30, −0.31

Computer programs: *APEX2* and *SAINT* (Bruker, 2012 ▸), *SHELXTL* (Sheldrick, 2008 ▸) and *SHELXL2013* (Sheldrick, 2015 ▸).

## supplementary crystallographic information

### Crystal data

**Table:**

C_23_H_18_N_3_O_3_^+^·CF_3_O_3_S^−^	*F*(000) = 2192
*M**_r_* = 533.47	*D*_x_ = 1.509 Mg m^−^^3^
Monoclinic, *P*2_1_/*c*	Mo *K*α radiation, λ = 0.71073 Å
*a* = 15.2183 (6) Å	Cell parameters from 9165 reflections
*b* = 20.0810 (8) Å	θ = 2.4–23.7°
*c* = 15.3652 (6) Å	µ = 0.21 mm^−^^1^
β = 90.544 (1)°	*T* = 298 K
*V* = 4695.4 (3) Å^3^	Prism, yellow
*Z* = 8	0.30 × 0.17 × 0.14 mm

### Data collection

**Table:**

Bruker APEXII CCD area-detector diffractometer	*R*_int_ = 0.095
Detector resolution: 0.83 pixels mm^-1^	θ_max_ = 25.4°, θ_min_ = 1.3°
ω scans	*h* = −18→18
38624 measured reflections	*k* = −24→24
8609 independent reflections	*l* = −17→18
3809 reflections with *I* > 2σ(*I*)	

### Refinement

**Table:**

Refinement on *F*^2^	180 restraints
Least-squares matrix: full	Hydrogen site location: mixed
*R*[*F*^2^ > 2σ(*F*^2^)] = 0.046	H atoms treated by a mixture of independent and constrained refinement
*wR*(*F*^2^) = 0.110	*w* = 1/[σ^2^(*F*_o_^2^) + (0.0366*P*)^2^] where *P* = (*F*_o_^2^ + 2*F*_c_^2^)/3
*S* = 0.85	(Δ/σ)_max_ < 0.001
8609 reflections	Δρ_max_ = 0.30 e Å^−^^3^
729 parameters	Δρ_min_ = −0.31 e Å^−^^3^

### Special details

**Table:**

Geometry. All esds (except the esd in the dihedral angle between two l.s. planes) are estimated using the full covariance matrix. The cell esds are taken into account individually in the estimation of esds in distances, angles and torsion angles; correlations between esds in cell parameters are only used when they are defined by crystal symmetry. An approximate (isotropic) treatment of cell esds is used for estimating esds involving l.s. planes.

### Fractional atomic coordinates and isotropic or equivalent isotropic displacement parameters (Å^2^)

**Table:**

	*x*	*y*	*z*	*U*_iso_*/*U*_eq_	Occ. (<1)
O1	0.41029 (15)	0.60296 (11)	0.34381 (13)	0.0614 (6)	
N2	0.1246 (2)	0.91541 (18)	0.5407 (2)	0.0720 (9)	
O2	0.05962 (19)	0.88908 (14)	0.50918 (19)	0.0895 (9)	
O3	0.1269 (2)	0.97245 (14)	0.5673 (2)	0.1157 (11)	
N1	0.42674 (16)	0.69242 (12)	0.57973 (16)	0.0455 (7)	
C2	0.42848 (19)	0.64777 (15)	0.51480 (19)	0.0465 (8)	
H2	0.4465	0.6611	0.4598	0.056*	
C3	0.40385 (19)	0.58199 (15)	0.52846 (19)	0.0422 (8)	
C4	0.37908 (19)	0.56351 (15)	0.61040 (19)	0.0463 (8)	
H4	0.3624	0.5197	0.6207	0.056*	
C5	0.3516 (2)	0.59151 (16)	0.7633 (2)	0.0599 (10)	
H5	0.3354	0.5478	0.7751	0.072*	
C6	0.3493 (2)	0.63777 (18)	0.8274 (2)	0.0686 (11)	
H6	0.3311	0.6257	0.8828	0.082*	
C7	0.3740 (2)	0.70292 (18)	0.8109 (2)	0.0668 (11)	
H7	0.3727	0.7338	0.8560	0.080*	
C8	0.4001 (2)	0.72291 (16)	0.7310 (2)	0.0581 (9)	
H8	0.4163	0.7669	0.7211	0.070*	
C9	0.4023 (2)	0.67572 (16)	0.66336 (19)	0.0466 (8)	
C10	0.3784 (2)	0.60934 (15)	0.67883 (19)	0.0453 (8)	
C11	0.4434 (2)	0.76315 (14)	0.55781 (19)	0.0535 (9)	
H11A	0.4713	0.7658	0.5014	0.064*	
H11B	0.4831	0.7824	0.6007	0.064*	
C12	0.3584 (2)	0.80241 (15)	0.55578 (18)	0.0450 (8)	
C13	0.2813 (2)	0.77440 (15)	0.52486 (19)	0.0522 (9)	
H13	0.2809	0.7301	0.5073	0.063*	
C14	0.2046 (2)	0.81107 (16)	0.51962 (19)	0.0550 (9)	
H14	0.1530	0.7921	0.4984	0.066*	
C15	0.2066 (2)	0.87608 (16)	0.5464 (2)	0.0511 (9)	
C16	0.2812 (3)	0.90518 (17)	0.5779 (2)	0.0627 (10)	
H16	0.2806	0.9492	0.5966	0.075*	
C17	0.3573 (2)	0.86843 (15)	0.5817 (2)	0.0599 (10)	
H17	0.4089	0.8882	0.6021	0.072*	
N18	0.40495 (17)	0.53477 (12)	0.46162 (16)	0.0457 (7)	
H18	0.3957 (19)	0.4931 (13)	0.4779 (18)	0.055*	
C19	0.4050 (2)	0.54681 (17)	0.3739 (2)	0.0492 (8)	
C20	0.4012 (2)	0.48600 (18)	0.3174 (2)	0.0520 (9)	
C21	0.3658 (2)	0.42642 (19)	0.3436 (2)	0.0667 (10)	
H21	0.3428	0.4223	0.3992	0.080*	
C22	0.3643 (3)	0.37257 (19)	0.2874 (3)	0.0831 (12)	
H22	0.3403	0.3324	0.3055	0.100*	
C23	0.3983 (3)	0.3781 (2)	0.2048 (3)	0.0891 (14)	
H23	0.3975	0.3418	0.1672	0.107*	
C24	0.4330 (3)	0.4374 (2)	0.1790 (2)	0.0874 (14)	
H24	0.4559	0.4415	0.1234	0.105*	
C25	0.4344 (2)	0.49175 (19)	0.2346 (2)	0.0700 (11)	
H25	0.4577	0.5321	0.2161	0.084*	
O21	0.20655 (15)	−0.02685 (11)	1.00566 (14)	0.0677 (7)	
N22	0.3601 (2)	0.43286 (16)	0.93943 (18)	0.0623 (8)	
O22	0.43183 (18)	0.40720 (13)	0.95183 (18)	0.0851 (8)	
O23	0.34872 (18)	0.49256 (13)	0.92938 (17)	0.0901 (9)	
N26	0.08024 (15)	0.19179 (11)	0.90797 (14)	0.0393 (6)	
C27	0.07780 (18)	0.14885 (14)	0.97427 (18)	0.0399 (7)	
H27	0.0608	0.1643	1.0286	0.048*	
C28	0.09965 (18)	0.08225 (14)	0.96521 (19)	0.0387 (7)	
C29	0.12306 (19)	0.05999 (14)	0.88465 (19)	0.0469 (8)	
H29	0.1370	0.0153	0.8768	0.056*	
C30	0.1490 (2)	0.08164 (17)	0.7297 (2)	0.0621 (10)	
H30	0.1614	0.0369	0.7202	0.074*	
C31	0.1528 (2)	0.12537 (18)	0.6631 (2)	0.0701 (11)	
H31	0.1677	0.1104	0.6079	0.084*	
C32	0.1348 (2)	0.19245 (18)	0.6759 (2)	0.0688 (11)	
H32	0.1390	0.2218	0.6293	0.083*	
C33	0.1111 (2)	0.21621 (16)	0.75568 (19)	0.0555 (9)	
H33	0.0986	0.2611	0.7635	0.067*	
C34	0.10618 (19)	0.17144 (15)	0.82524 (18)	0.0420 (8)	
C35	0.12631 (19)	0.10371 (15)	0.81382 (19)	0.0439 (8)	
C36	0.05804 (19)	0.26267 (13)	0.92396 (18)	0.0452 (8)	
H36A	0.0277	0.2663	0.9790	0.054*	
H36B	0.0184	0.2780	0.8784	0.054*	
C37	0.1383 (2)	0.30700 (15)	0.92630 (17)	0.0406 (8)	
C38	0.2186 (2)	0.28329 (15)	0.95501 (19)	0.0498 (9)	
H38	0.2239	0.2389	0.9713	0.060*	
C39	0.2909 (2)	0.32433 (16)	0.95987 (19)	0.0533 (9)	
H39	0.3449	0.3080	0.9791	0.064*	
C40	0.2817 (2)	0.38968 (16)	0.93586 (19)	0.0469 (8)	
C41	0.2032 (2)	0.41526 (15)	0.90788 (19)	0.0538 (9)	
H41	0.1984	0.4600	0.8927	0.065*	
C42	0.1312 (2)	0.37365 (15)	0.90253 (19)	0.0500 (9)	
H42	0.0776	0.3903	0.8829	0.060*	
C43	0.1481 (2)	−0.01105 (16)	1.0550 (2)	0.0490 (8)	
N44	0.09404 (17)	0.04176 (12)	1.03937 (16)	0.0437 (7)	
H44	0.0575 (18)	0.0565 (13)	1.0780 (17)	0.052*	
C45	0.1292 (2)	−0.04973 (15)	1.1360 (2)	0.0475 (8)	
C46	0.1440 (2)	−0.11755 (17)	1.1338 (2)	0.0683 (10)	
H46	0.1655	−0.1371	1.0835	0.082*	
C47	0.1271 (3)	−0.15649 (19)	1.2059 (3)	0.0836 (13)	
H47	0.1361	−0.2023	1.2035	0.100*	
C48	0.0972 (3)	−0.1277 (2)	1.2808 (3)	0.0844 (13)	
H48	0.0865	−0.1540	1.3294	0.101*	
C49	0.0831 (2)	−0.0606 (2)	1.2844 (2)	0.0726 (11)	
H49	0.0632	−0.0412	1.3356	0.087*	
C50	0.0984 (2)	−0.02119 (16)	1.2121 (2)	0.0587 (9)	
H50	0.0881	0.0244	1.2146	0.070*	
S1	0.35061 (6)	0.14986 (4)	0.08683 (6)	0.0579 (3)	
O4	0.39929 (14)	0.09068 (10)	0.06530 (13)	0.0613 (6)	
O5	0.39719 (15)	0.21137 (10)	0.07281 (15)	0.0781 (8)	
O6	0.25996 (16)	0.14934 (12)	0.06060 (18)	0.0973 (9)	
C51	0.3430 (3)	0.14538 (19)	0.2041 (3)	0.0859 (12)	
F1	0.4184 (6)	0.1409 (9)	0.2480 (7)	0.116 (3)	0.56 (3)
F2	0.2924 (10)	0.0910 (5)	0.2186 (10)	0.129 (3)	0.56 (3)
F3	0.2872 (10)	0.1946 (7)	0.2229 (12)	0.133 (4)	0.56 (3)
F1A	0.4226 (7)	0.1671 (10)	0.2312 (13)	0.127 (4)	0.44 (3)
F2A	0.3289 (15)	0.0856 (4)	0.2381 (9)	0.108 (4)	0.44 (3)
F3A	0.3015 (13)	0.1924 (7)	0.2490 (10)	0.099 (3)	0.44 (3)
S2	0.92877 (6)	0.15447 (4)	0.17934 (6)	0.0565 (3)	
O24	0.95479 (15)	0.09446 (11)	0.13662 (16)	0.0838 (8)	
O25	0.94245 (17)	0.21354 (10)	0.12816 (15)	0.0827 (8)	
O26	0.95282 (18)	0.15957 (13)	0.26900 (16)	0.1016 (9)	
C52	0.8106 (2)	0.14561 (15)	0.1834 (2)	0.0563 (9)	
F21	0.7755 (4)	0.1902 (3)	0.2367 (6)	0.0916 (17)	0.83 (2)
F22	0.7851 (5)	0.0871 (3)	0.2147 (5)	0.0707 (15)	0.83 (2)
F23	0.7716 (4)	0.1511 (4)	0.1065 (3)	0.0856 (16)	0.83 (2)
F21A	0.7749 (17)	0.2051 (8)	0.196 (2)	0.080 (5)	0.17 (2)
F22A	0.807 (3)	0.0919 (13)	0.234 (2)	0.081 (6)	0.17 (2)
F23A	0.800 (3)	0.1368 (16)	0.0978 (7)	0.090 (5)	0.17 (2)

### Atomic displacement parameters (Å^2^)

**Table:**

	*U*^11^	*U*^22^	*U*^33^	*U*^12^	*U*^13^	*U*^23^
O1	0.0661 (16)	0.0638 (16)	0.0543 (15)	0.0036 (14)	−0.0066 (12)	0.0038 (13)
N2	0.080 (3)	0.064 (2)	0.072 (2)	0.015 (2)	0.013 (2)	0.0031 (19)
O2	0.072 (2)	0.093 (2)	0.104 (2)	0.0111 (18)	0.0038 (18)	0.0070 (17)
O3	0.121 (3)	0.073 (2)	0.153 (3)	0.037 (2)	−0.005 (2)	−0.026 (2)
N1	0.0465 (17)	0.0428 (16)	0.0471 (16)	0.0001 (13)	0.0048 (14)	−0.0032 (14)
C2	0.046 (2)	0.048 (2)	0.046 (2)	0.0048 (17)	0.0087 (16)	−0.0044 (17)
C3	0.0352 (18)	0.050 (2)	0.0412 (19)	0.0054 (16)	0.0001 (16)	−0.0056 (17)
C4	0.047 (2)	0.045 (2)	0.047 (2)	0.0002 (16)	0.0000 (17)	−0.0012 (17)
C5	0.077 (3)	0.056 (2)	0.046 (2)	0.009 (2)	0.0049 (19)	0.0014 (19)
C6	0.095 (3)	0.070 (3)	0.041 (2)	0.014 (2)	0.009 (2)	−0.002 (2)
C7	0.096 (3)	0.060 (3)	0.045 (2)	0.015 (2)	−0.003 (2)	−0.0100 (19)
C8	0.069 (3)	0.055 (2)	0.051 (2)	0.0067 (19)	−0.003 (2)	−0.0096 (19)
C9	0.046 (2)	0.054 (2)	0.041 (2)	0.0075 (17)	−0.0020 (16)	−0.0048 (17)
C10	0.047 (2)	0.048 (2)	0.0413 (19)	0.0050 (17)	0.0009 (16)	−0.0039 (17)
C11	0.056 (2)	0.048 (2)	0.057 (2)	−0.0104 (18)	0.0088 (18)	−0.0081 (17)
C12	0.054 (2)	0.041 (2)	0.0406 (19)	−0.0049 (18)	0.0108 (17)	−0.0020 (15)
C13	0.065 (2)	0.039 (2)	0.053 (2)	−0.0030 (19)	−0.0014 (19)	−0.0063 (16)
C14	0.056 (2)	0.051 (2)	0.058 (2)	−0.006 (2)	−0.0022 (19)	−0.0012 (18)
C15	0.061 (2)	0.046 (2)	0.046 (2)	0.004 (2)	0.0093 (19)	0.0023 (17)
C16	0.076 (3)	0.045 (2)	0.067 (2)	0.000 (2)	0.013 (2)	−0.0120 (19)
C17	0.064 (3)	0.048 (2)	0.069 (2)	−0.010 (2)	0.009 (2)	−0.0169 (18)
N18	0.0521 (17)	0.0449 (16)	0.0403 (17)	−0.0001 (15)	0.0034 (14)	−0.0053 (15)
C19	0.0376 (19)	0.061 (2)	0.049 (2)	0.0065 (19)	−0.0018 (17)	−0.004 (2)
C20	0.046 (2)	0.070 (3)	0.040 (2)	0.014 (2)	−0.0078 (17)	−0.0092 (19)
C21	0.066 (3)	0.078 (3)	0.055 (2)	−0.006 (2)	0.000 (2)	−0.019 (2)
C22	0.086 (3)	0.081 (3)	0.083 (3)	−0.005 (2)	−0.014 (3)	−0.029 (2)
C23	0.087 (3)	0.107 (4)	0.072 (3)	0.026 (3)	−0.023 (3)	−0.045 (3)
C24	0.092 (3)	0.122 (4)	0.049 (3)	0.027 (3)	−0.009 (2)	−0.021 (3)
C25	0.076 (3)	0.088 (3)	0.045 (2)	0.017 (2)	−0.004 (2)	−0.003 (2)
O21	0.0592 (16)	0.0751 (17)	0.0689 (16)	0.0210 (14)	0.0073 (13)	0.0066 (13)
N22	0.075 (2)	0.055 (2)	0.0571 (19)	−0.009 (2)	0.0089 (19)	−0.0023 (16)
O22	0.0620 (18)	0.082 (2)	0.112 (2)	−0.0055 (16)	−0.0009 (17)	−0.0033 (16)
O23	0.103 (2)	0.0574 (17)	0.110 (2)	−0.0216 (17)	0.0022 (17)	0.0036 (16)
N26	0.0430 (16)	0.0368 (15)	0.0381 (15)	0.0024 (12)	0.0004 (13)	−0.0012 (12)
C27	0.0421 (19)	0.044 (2)	0.0331 (17)	−0.0033 (16)	0.0004 (15)	0.0026 (16)
C28	0.0378 (18)	0.0353 (19)	0.0429 (19)	−0.0011 (15)	−0.0015 (15)	0.0035 (16)
C29	0.054 (2)	0.0348 (19)	0.052 (2)	−0.0007 (16)	−0.0025 (18)	−0.0033 (17)
C30	0.083 (3)	0.053 (2)	0.050 (2)	0.009 (2)	0.001 (2)	−0.0102 (19)
C31	0.094 (3)	0.074 (3)	0.043 (2)	0.015 (2)	0.007 (2)	−0.007 (2)
C32	0.094 (3)	0.073 (3)	0.039 (2)	0.011 (2)	0.008 (2)	0.0087 (19)
C33	0.073 (3)	0.053 (2)	0.041 (2)	0.0073 (19)	−0.0007 (19)	0.0064 (17)
C34	0.0430 (19)	0.047 (2)	0.0364 (18)	0.0009 (16)	−0.0011 (15)	−0.0009 (16)
C35	0.045 (2)	0.047 (2)	0.0404 (19)	0.0004 (17)	−0.0012 (16)	−0.0042 (17)
C36	0.051 (2)	0.0386 (19)	0.0461 (19)	0.0099 (16)	0.0063 (17)	0.0004 (15)
C37	0.050 (2)	0.041 (2)	0.0308 (17)	0.0041 (17)	0.0047 (16)	0.0014 (15)
C38	0.059 (2)	0.0348 (19)	0.055 (2)	0.0038 (18)	−0.0005 (19)	0.0087 (16)
C39	0.054 (2)	0.053 (2)	0.052 (2)	0.0070 (19)	−0.0035 (18)	0.0078 (18)
C40	0.054 (2)	0.046 (2)	0.0403 (19)	−0.0052 (19)	0.0021 (17)	0.0006 (16)
C41	0.065 (2)	0.038 (2)	0.058 (2)	0.002 (2)	0.008 (2)	0.0099 (17)
C42	0.055 (2)	0.044 (2)	0.052 (2)	0.0122 (18)	−0.0015 (18)	0.0088 (16)
C43	0.042 (2)	0.050 (2)	0.055 (2)	−0.0022 (18)	−0.0071 (18)	0.0008 (18)
N44	0.0464 (17)	0.0386 (16)	0.0460 (17)	0.0048 (14)	0.0057 (13)	0.0049 (14)
C45	0.042 (2)	0.041 (2)	0.059 (2)	−0.0010 (16)	−0.0108 (17)	0.0125 (18)
C46	0.071 (3)	0.047 (2)	0.086 (3)	0.004 (2)	−0.007 (2)	0.009 (2)
C47	0.085 (3)	0.049 (2)	0.116 (4)	−0.010 (2)	−0.028 (3)	0.031 (3)
C48	0.082 (3)	0.088 (4)	0.083 (3)	−0.024 (3)	−0.024 (3)	0.035 (3)
C49	0.078 (3)	0.079 (3)	0.060 (3)	−0.010 (2)	−0.012 (2)	0.015 (2)
C50	0.069 (3)	0.051 (2)	0.056 (2)	−0.0022 (19)	−0.012 (2)	0.0121 (19)
S1	0.0589 (6)	0.0519 (6)	0.0629 (6)	0.0021 (5)	0.0004 (5)	0.0137 (5)
O4	0.0767 (17)	0.0447 (14)	0.0624 (14)	0.0070 (13)	0.0043 (13)	−0.0032 (11)
O5	0.0778 (18)	0.0469 (15)	0.110 (2)	−0.0095 (13)	0.0207 (15)	0.0183 (13)
O6	0.0554 (17)	0.094 (2)	0.142 (2)	−0.0019 (16)	−0.0331 (17)	0.0289 (18)
C51	0.097 (3)	0.084 (3)	0.078 (3)	0.030 (2)	0.031 (2)	0.015 (2)
F1	0.149 (4)	0.152 (8)	0.047 (3)	0.048 (4)	−0.006 (3)	0.021 (4)
F2	0.148 (7)	0.122 (4)	0.120 (7)	0.011 (4)	0.062 (5)	0.064 (4)
F3	0.159 (6)	0.144 (4)	0.097 (8)	0.079 (4)	0.046 (6)	−0.011 (5)
F1A	0.132 (4)	0.150 (9)	0.098 (8)	0.025 (4)	−0.027 (5)	−0.011 (6)
F2A	0.183 (9)	0.088 (4)	0.053 (5)	0.042 (4)	0.021 (6)	0.033 (3)
F3A	0.150 (6)	0.100 (4)	0.047 (5)	0.040 (5)	0.026 (5)	0.006 (4)
S2	0.0512 (6)	0.0511 (6)	0.0671 (6)	−0.0032 (5)	0.0020 (5)	−0.0055 (5)
O24	0.0663 (17)	0.0554 (15)	0.130 (2)	0.0093 (13)	0.0378 (16)	−0.0202 (15)
O25	0.097 (2)	0.0514 (15)	0.1002 (19)	−0.0166 (14)	0.0260 (16)	0.0076 (14)
O26	0.098 (2)	0.131 (2)	0.0753 (19)	−0.0293 (19)	−0.0394 (16)	0.0004 (17)
C52	0.058 (2)	0.050 (2)	0.060 (2)	0.0106 (19)	0.0017 (19)	−0.0027 (18)
F21	0.088 (2)	0.075 (2)	0.112 (4)	0.0202 (19)	0.040 (3)	−0.014 (3)
F22	0.054 (3)	0.0696 (18)	0.089 (3)	−0.0102 (18)	0.002 (2)	0.015 (2)
F23	0.056 (3)	0.113 (4)	0.0874 (19)	0.013 (2)	−0.0224 (19)	0.0210 (19)
F21A	0.059 (8)	0.079 (6)	0.102 (10)	0.035 (7)	0.002 (9)	−0.008 (6)
F22A	0.073 (13)	0.068 (7)	0.103 (10)	−0.022 (8)	0.014 (10)	0.017 (7)
F23A	0.086 (13)	0.105 (10)	0.079 (5)	0.037 (10)	−0.053 (8)	−0.019 (6)

### Geometric parameters (Å, º)

**Table:**

O1—C19	1.222 (3)	C28—N44	1.403 (3)
N2—O3	1.217 (3)	C29—C35	1.400 (4)
N2—O2	1.218 (4)	C29—H29	0.9300
N2—C15	1.478 (4)	C30—C31	1.351 (4)
N1—C2	1.342 (3)	C30—C35	1.412 (4)
N1—C9	1.383 (3)	C30—H30	0.9300
N1—C11	1.482 (3)	C31—C32	1.389 (4)
C2—C3	1.390 (4)	C31—H31	0.9300
C2—H2	0.9300	C32—C33	1.366 (4)
C3—C4	1.369 (4)	C32—H32	0.9300
C3—N18	1.398 (3)	C33—C34	1.399 (4)
C4—C10	1.397 (4)	C33—H33	0.9300
C4—H4	0.9300	C34—C35	1.405 (4)
C5—C6	1.355 (4)	C36—C37	1.511 (4)
C5—C10	1.411 (4)	C36—H36A	0.9700
C5—H5	0.9300	C36—H36B	0.9700
C6—C7	1.385 (4)	C37—C38	1.380 (4)
C6—H6	0.9300	C37—C42	1.391 (4)
C7—C8	1.355 (4)	C38—C39	1.376 (4)
C7—H7	0.9300	C38—H38	0.9300
C8—C9	1.408 (4)	C39—C40	1.370 (4)
C8—H8	0.9300	C39—H39	0.9300
C9—C10	1.402 (4)	C40—C41	1.366 (4)
C11—C12	1.516 (4)	C41—C42	1.380 (4)
C11—H11A	0.9700	C41—H41	0.9300
C11—H11B	0.9700	C42—H42	0.9300
C12—C13	1.381 (4)	C43—N44	1.362 (4)
C12—C17	1.385 (4)	C43—C45	1.497 (4)
C13—C14	1.381 (4)	N44—H44	0.87 (2)
C13—H13	0.9300	C45—C46	1.381 (4)
C14—C15	1.369 (4)	C45—C50	1.387 (4)
C14—H14	0.9300	C46—C47	1.381 (5)
C15—C16	1.362 (4)	C46—H46	0.9300
C16—C17	1.374 (4)	C47—C48	1.370 (5)
C16—H16	0.9300	C47—H47	0.9300
C17—H17	0.9300	C48—C49	1.366 (5)
N18—C19	1.369 (4)	C48—H48	0.9300
N18—H18	0.88 (3)	C49—C50	1.385 (4)
C19—C20	1.499 (4)	C49—H49	0.9300
C20—C21	1.374 (4)	C50—H50	0.9300
C20—C25	1.378 (4)	S1—O6	1.434 (2)
C21—C22	1.383 (4)	S1—O4	1.441 (2)
C21—H21	0.9300	S1—O5	1.441 (2)
C22—C23	1.380 (5)	S1—C51	1.809 (4)
C22—H22	0.9300	C51—F2A	1.326 (7)
C23—C24	1.364 (5)	C51—F1	1.328 (6)
C23—H23	0.9300	C51—F3A	1.332 (7)
C24—C25	1.386 (5)	C51—F3	1.337 (6)
C24—H24	0.9300	C51—F1A	1.350 (7)
C25—H25	0.9300	C51—F2	1.355 (6)
O21—C43	1.217 (3)	S2—O26	1.426 (2)
N22—O22	1.221 (3)	S2—O24	1.430 (2)
N22—O23	1.221 (3)	S2—O25	1.439 (2)
N22—C40	1.475 (4)	S2—C52	1.809 (4)
N26—C27	1.335 (3)	C52—F23	1.321 (4)
N26—C34	1.396 (3)	C52—F21A	1.329 (8)
N26—C36	1.484 (3)	C52—F22A	1.329 (8)
C27—C28	1.386 (4)	C52—F22	1.330 (4)
C27—H27	0.9300	C52—F21	1.330 (4)
C28—C29	1.366 (4)	C52—F23A	1.335 (8)
			
O3—N2—O2	124.2 (4)	C30—C31—C32	120.8 (3)
O3—N2—C15	117.5 (4)	C30—C31—H31	119.6
O2—N2—C15	118.3 (3)	C32—C31—H31	119.6
C2—N1—C9	122.4 (3)	C33—C32—C31	121.4 (3)
C2—N1—C11	117.8 (2)	C33—C32—H32	119.3
C9—N1—C11	119.5 (2)	C31—C32—H32	119.3
N1—C2—C3	121.0 (3)	C32—C33—C34	118.5 (3)
N1—C2—H2	119.5	C32—C33—H33	120.7
C3—C2—H2	119.5	C34—C33—H33	120.7
C4—C3—C2	118.3 (3)	N26—C34—C33	121.7 (3)
C4—C3—N18	119.8 (3)	N26—C34—C35	117.5 (3)
C2—C3—N18	121.9 (3)	C33—C34—C35	120.9 (3)
C3—C4—C10	121.2 (3)	C29—C35—C34	120.0 (3)
C3—C4—H4	119.4	C29—C35—C30	121.7 (3)
C10—C4—H4	119.4	C34—C35—C30	118.3 (3)
C6—C5—C10	120.3 (3)	N26—C36—C37	112.6 (2)
C6—C5—H5	119.8	N26—C36—H36A	109.1
C10—C5—H5	119.8	C37—C36—H36A	109.1
C5—C6—C7	120.4 (3)	N26—C36—H36B	109.1
C5—C6—H6	119.8	C37—C36—H36B	109.1
C7—C6—H6	119.8	H36A—C36—H36B	107.8
C8—C7—C6	121.9 (3)	C38—C37—C42	118.8 (3)
C8—C7—H7	119.0	C38—C37—C36	121.1 (3)
C6—C7—H7	119.0	C42—C37—C36	120.0 (3)
C7—C8—C9	118.6 (3)	C39—C38—C37	121.1 (3)
C7—C8—H8	120.7	C39—C38—H38	119.5
C9—C8—H8	120.7	C37—C38—H38	119.5
N1—C9—C10	117.4 (3)	C40—C39—C38	118.7 (3)
N1—C9—C8	122.1 (3)	C40—C39—H39	120.7
C10—C9—C8	120.5 (3)	C38—C39—H39	120.7
C4—C10—C9	119.7 (3)	C41—C40—C39	122.1 (3)
C4—C10—C5	122.0 (3)	C41—C40—N22	119.7 (3)
C9—C10—C5	118.3 (3)	C39—C40—N22	118.2 (3)
N1—C11—C12	110.8 (2)	C40—C41—C42	118.8 (3)
N1—C11—H11A	109.5	C40—C41—H41	120.6
C12—C11—H11A	109.5	C42—C41—H41	120.6
N1—C11—H11B	109.5	C41—C42—C37	120.5 (3)
C12—C11—H11B	109.5	C41—C42—H42	119.7
H11A—C11—H11B	108.1	C37—C42—H42	119.7
C13—C12—C17	118.5 (3)	O21—C43—N44	122.5 (3)
C13—C12—C11	121.2 (3)	O21—C43—C45	122.0 (3)
C17—C12—C11	120.3 (3)	N44—C43—C45	115.5 (3)
C14—C13—C12	121.1 (3)	C43—N44—C28	123.6 (3)
C14—C13—H13	119.4	C43—N44—H44	122.2 (19)
C12—C13—H13	119.4	C28—N44—H44	113.7 (19)
C15—C14—C13	118.4 (3)	C46—C45—C50	119.0 (3)
C15—C14—H14	120.8	C46—C45—C43	117.3 (3)
C13—C14—H14	120.8	C50—C45—C43	123.7 (3)
C16—C15—C14	122.1 (3)	C47—C46—C45	120.5 (4)
C16—C15—N2	119.5 (3)	C47—C46—H46	119.8
C14—C15—N2	118.4 (4)	C45—C46—H46	119.8
C15—C16—C17	119.0 (3)	C48—C47—C46	120.0 (4)
C15—C16—H16	120.5	C48—C47—H47	120.0
C17—C16—H16	120.5	C46—C47—H47	120.0
C16—C17—C12	120.9 (3)	C49—C48—C47	120.2 (4)
C16—C17—H17	119.5	C49—C48—H48	119.9
C12—C17—H17	119.5	C47—C48—H48	119.9
C19—N18—C3	127.1 (3)	C48—C49—C50	120.2 (4)
C19—N18—H18	116.6 (19)	C48—C49—H49	119.9
C3—N18—H18	115.5 (18)	C50—C49—H49	119.9
O1—C19—N18	122.4 (3)	C49—C50—C45	120.1 (3)
O1—C19—C20	122.3 (3)	C49—C50—H50	120.0
N18—C19—C20	115.2 (3)	C45—C50—H50	120.0
C21—C20—C25	119.4 (3)	O6—S1—O4	115.16 (15)
C21—C20—C19	123.6 (3)	O6—S1—O5	115.95 (15)
C25—C20—C19	117.0 (3)	O4—S1—O5	114.69 (13)
C20—C21—C22	120.2 (3)	O6—S1—C51	102.08 (18)
C20—C21—H21	119.9	O4—S1—C51	103.03 (15)
C22—C21—H21	119.9	O5—S1—C51	103.16 (17)
C23—C22—C21	120.4 (4)	F2A—C51—F3A	111.0 (11)
C23—C22—H22	119.8	F1—C51—F3	119.1 (14)
C21—C22—H22	119.8	F2A—C51—F1A	108.6 (8)
C24—C23—C22	119.1 (4)	F3A—C51—F1A	92.3 (13)
C24—C23—H23	120.4	F1—C51—F2	110.5 (6)
C22—C23—H23	120.4	F3—C51—F2	101.4 (10)
C23—C24—C25	120.8 (4)	F2A—C51—S1	116.7 (7)
C23—C24—H24	119.6	F1—C51—S1	116.5 (6)
C25—C24—H24	119.6	F3A—C51—S1	121.0 (8)
C20—C25—C24	120.0 (4)	F3—C51—S1	103.0 (8)
C20—C25—H25	120.0	F1A—C51—S1	103.0 (9)
C24—C25—H25	120.0	F2—C51—S1	104.3 (7)
O22—N22—O23	124.0 (3)	O26—S2—O24	115.75 (17)
O22—N22—C40	118.6 (3)	O26—S2—O25	115.56 (16)
O23—N22—C40	117.3 (3)	O24—S2—O25	113.67 (14)
C27—N26—C34	121.0 (2)	O26—S2—C52	102.72 (16)
C27—N26—C36	119.0 (2)	O24—S2—C52	102.28 (14)
C34—N26—C36	119.9 (2)	O25—S2—C52	104.40 (16)
N26—C27—C28	122.6 (3)	F21A—C52—F22A	128 (3)
N26—C27—H27	118.7	F23—C52—F22	105.5 (5)
C28—C27—H27	118.7	F23—C52—F21	108.4 (4)
C29—C28—C27	118.1 (3)	F22—C52—F21	104.6 (5)
C29—C28—N44	124.4 (3)	F21A—C52—F23A	102.8 (15)
C27—C28—N44	117.5 (3)	F22A—C52—F23A	117 (2)
C28—C29—C35	120.7 (3)	F23—C52—S2	113.6 (4)
C28—C29—H29	119.6	F21A—C52—S2	108.9 (13)
C35—C29—H29	119.6	F22A—C52—S2	98.7 (18)
C31—C30—C35	120.2 (3)	F22—C52—S2	113.1 (4)
C31—C30—H30	119.9	F21—C52—S2	111.1 (3)
C35—C30—H30	119.9	F23A—C52—S2	95.3 (17)

### Hydrogen-bond geometry (Å, º)

**Table:**

*D*—H···*A*	*D*—H	H···*A*	*D*···*A*	*D*—H···*A*
N18—H18···O4^i^	0.88 (3)	2.15 (3)	2.982 (3)	156 (3)
N44—H44···O24^ii^	0.87 (2)	1.97 (3)	2.811 (3)	164 (3)

Symmetry codes: (i) *x*, −*y*+1/2, *z*+1/2; (ii) *x*−1, *y*, *z*+1.
